# An international reproducibility study validating quantitative determination of *ERBB2*, *ESR1*, *PGR*, and *MKI67* mRNA in breast cancer using MammaTyper®

**DOI:** 10.1186/s13058-017-0848-z

**Published:** 2017-05-11

**Authors:** Zsuzsanna Varga, Annette Lebeau, Hong Bu, Arndt Hartmann, Frederique Penault-Llorca, Elena Guerini-Rocco, Peter Schraml, Fraser Symmans, Robert Stoehr, Xiaodong Teng, Andreas Turzynski, Reinhard von Wasielewski, Claudia Gürtler, Mark Laible, Kornelia Schlombs, Heikki Joensuu, Thomas Keller, Peter Sinn, Ugur Sahin, John Bartlett, Giuseppe Viale

**Affiliations:** 1 Institute of Pathology and Molecular Pathology, University Hospital Zurich, Zurich, Switzerland; 2 Private Group Practice for Pathology and PathoPlan GbR, Lübeck, Germany; 3 Department of Pathology and Laboratory of Pathology, West China Hospital, Sichuan University, Chengdu, China; 4 Institute of Pathology, University Erlangen-Nürnberg, Erlangen, Germany; 5 Centre Jean Perrin, Centre regional de Lutte contre le cancer d’Auvergne, Clermont-Ferrand, France; 6 European Institute of Oncology, University of Milan, Milan, Italy; 7 University of Texas MD Anderson Cancer Center, Houston, TX USA; 8 Department of Pathology, The First Affiliated Hospital, School of Medicine, Zhejiang University, Hangzhou, China; 9 Institute of Pathology, Klinikum Region Hannover, Hannover, Germany; 10 BioNTech Diagnostics GmbH, Mainz, Germany; 11 Department of Oncology, Helsinki University Hospital and University of Helsinki, Helsinki, Finland; 12 ACOMED Statistik, Leipzig, Germany; 13 Department of Pathology, Heidelberg University Hospital, Heidelberg, Germany; 14 Transformative Pathology, Ontario Institute for Cancer Research (OICR), Toronto, ON Canada

**Keywords:** MammaTyper, Reproducibility, RT-qPCR, FFPE, Breast cancer, ERBB2, ESR1, PGR, MKI67

## Abstract

**Background:**

Accurate determination of the predictive markers human epidermal growth factor receptor 2 (HER2/*ERBB2*), estrogen receptor (ER/*ESR1*), progesterone receptor (PgR/*PGR*), and marker of proliferation Ki67 (*MKI67*) is indispensable for therapeutic decision making in early breast cancer. In this multicenter prospective study, we addressed the issue of inter- and intrasite reproducibility using the recently developed reverse transcription-quantitative real-time polymerase chain reaction-based MammaTyper® test.

**Methods:**

Ten international pathology institutions participated in this study and determined messenger RNA expression levels of *ERBB2*, *ESR1*, *PGR*, and *MKI67* in both centrally and locally extracted RNA from formalin-fixed, paraffin-embedded breast cancer specimens with the MammaTyper® test. Samples were measured repeatedly on different days within the local laboratories, and reproducibility was assessed by means of variance component analysis, Fleiss’ kappa statistics, and interclass correlation coefficients (ICCs).

**Results:**

Total variations in measurements of centrally and locally prepared RNA extracts were comparable; therefore, statistical analyses were performed on the complete dataset. Intersite reproducibility showed total SDs between 0.21 and 0.44 for the quantitative single-marker assessments, resulting in ICC values of 0.980–0.998, demonstrating excellent agreement of quantitative measurements. Also, the reproducibility of binary single-marker results (positive/negative), as well as the molecular subtype agreement, was almost perfect with kappa values ranging from 0.90 to 1.00.

**Conclusions:**

On the basis of these data, the MammaTyper® has the potential to substantially improve the current standards of breast cancer diagnostics by providing a highly precise and reproducible quantitative assessment of the established breast cancer biomarkers and molecular subtypes in a decentralized workup.

**Electronic supplementary material:**

The online version of this article (doi:10.1186/s13058-017-0848-z) contains supplementary material, which is available to authorized users.

## Background

In contemporary clinical management of patients with breast cancer, prognostications and therapeutic decisions are based on the assessment of clinicopathological factors as well as on the expression status of biomarkers with established clinical validity (i.e., human epidermal growth factor receptor 2 [HER2]; estrogen receptor [ER]; progesterone receptor [PgR]; and Ki67, a marker of cell proliferation) [1, 2]. Currently, the most commonly applied method for the determination of these four markers is immunohistochemistry (IHC), which allows for the semiquantitative assessment of the protein expression levels on histological slides [3, 4]. For HER2, an additional analysis of the amplification status of the corresponding gene *ERBB2* by fluorescence in situ hybridization (FISH), chromogenic in situ hybridization (CISH), or silver in situ hybridization (SISH) can also be applied in selected cases. The quality of the determination of these markers in terms of accuracy and reproducibility is essential for effective therapeutic interventions. However, the inter- and intraobserver variability of IHC is of concern [3–9]. For HER2, ER, and PgR, several studies have reported discrepancies of up to 20% [5–7], but most prominent and challenging is the inconsistency regarding Ki67 [8, 9]. Ki67 is a marker of the proliferative activity of the tumor cells and thereby carries valuable prognostic information [10–12]. In addition, Ki67 may have a direct impact on therapeutic decisions by assisting in the distinction between luminal A and luminal B breast cancer and therefore may aid in the selection of cytotoxic chemotherapy in addition to endocrine treatment [2, 13]. The variability in Ki67 is due mainly to the subjectivity of the visual estimation method and the choice of areas of evaluation on the histological slides and, to a lesser extent, the technical variations in the IHC staining process [9, 14]. Efforts to standardize Ki67 scoring resulted in considerable improvements, but interobserver agreement is still unsatisfactory [15, 16]. In addition, implementation of these methodological advances in clinical routine laboratories is challenging, and clinical validity of the new methods remains to be shown. For these reasons, the Ki67 determined by IHC is not currently included in the American Society of Clinical Oncology/College of American Pathologists guidelines for routine clinical use [1, 17]. There remains an urgent need for alternative, more robust, standardized, and precise assays with proven analytical and clinical validity for Ki67, HER2, ER, and PgR in routine breast cancer diagnostics [17, 18].

The MammaTyper® (BioNTech Diagnostics, Mainz, Germany) is a novel CE-marked in vitro diagnostic test that quantifies the messenger RNA (mRNA) expression of the four key marker genes *ERBB2*, *ESR1*, *PGR*, and *MKI67* on the basis of reverse transcription-quantitative real-time polymerase chain reaction (RT-qPCR), which differs from the currently applied standard of protein-based semiquantitative assessment by IHC. The main goal in using this technology is to provide a precise and reproducible assessment of the four biomarkers. Similarly to IHC, the MammaTyper® test can be integrated into the local laboratory setup because it supports analysis on widely accessible qPCR platforms using total RNA extracted from clinical routine formalin-fixed, paraffin-embedded (FFPE) breast cancer samples from resections or core needle biopsies.

In this study, we assessed the precision of the MammaTyper® test with a focus on reproducibility [19]. We adopted a multicenter design to fully evaluate the inter- and intrasite components of precision as well as other sources of imprecision, including preanalytical factors. Ten international pathology institutions, all with expert-level background in the field of breast cancer diagnostics, participated in the study. Each site carried out the same technical procedures according to a predefined study plan based on the EP05-A3 guideline for precision evaluation of quantitative measurement methods issued by the Clinical & Laboratory Standards Institute [20]. To our knowledge, a similar study has not been conducted to date.

## Methods

### Study objectives

The precision (reproducibility) of the MammaTyper® test was evaluated on multiple levels according to the following parameter definitions:

1. Intermediate precision, here also referred as *interrun precision*, as the variability of quantitative results across repeated measurements over several days by the same operator, in the same laboratory, and using the same instrument; this parameter also included repeatability, the variance component due to simple replicates (intrarun)
2. Intersite reproducibility, as the most comprehensive demonstration of precision, including the variability introduced by different laboratories, operators, and instruments
3. Preanalytical and lot-to-lot variability
4. Agreement of binary single-marker results and subtypes
5. Interclass correlation coefficient (ICC) as the agreement of quantitative results

### Study design

**Fig. 1:**
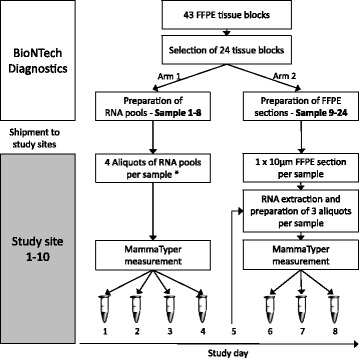
Study design. *One site measured additionally a second set of samples 1–8 using a second lot of MammaTyper®. *FFPE* Formalin-fixed, paraffin-embedded

A prospective, two-stage study was designed with the participation of ten international pathology institutions (*see* authors’ affiliations 1–9 and 14). Prior to the study start, one operator per site was trained on the correct use of the preanalytical RNA extraction kit RNXtract® (BioNTech Diagnostics) and the MammaTyper® test within a 2-day standard training phase carried out by the manufacturer. This training also included qualification of the local qPCR instrument for use with the MammaTyper®, which in this study was the LightCycler® 480 instrument II (Roche Molecular Diagnostics, Pleasanton, CA, USA). The training was followed by a familiarization period consisting of at least four MammaTyper® runs on 3–4 days using BioNTech Diagnostics’ reference material, carried out by the operator without supervision. During the study, each site performed repeated MammaTyper® measurements on different days according to a predefined study plan using RNA extracts from clinical FFPE breast cancer tissues. The same MammaTyper® lot was used at all sites, and only one site repeated study arm 1 using a second lot of MammaTyper®. The study comprised 8 days in total (consecutive or nonconsecutive days), as illustrated in the study design (Fig. 1).

#### Study arm 1

RNA was extracted at a central laboratory (BioNTech Diagnostics), and eight different RNA pools, each containing RNA from a single tumor sample, were provided as single-use aliquots to the study sites (samples 1–8). Samples were measured repeatedly on 4 different days using MammaTyper®.

#### Study arm 2

Ten-micrometer sections of 16 FFPE tissue samples from different breast tumors (samples 9–24) were provided to each study site for local RNA extraction using RNXtract®. After extraction, each RNA eluate was split into three single-use aliquots for repeated MammaTyper® measurements on 3 different days.

### Samples

The samples used in the study were prepared from clinical FFPE breast cancer tissue blocks by BioNTech Diagnostics and were distributed to study sites as RNA aliquots (samples 1–8) or 10-μm FFPE whole-tissue sections (samples 9–24). The 24 FFPE tissue samples were selected from a series of clinical routine breast cancer cases (*n* = 43) kindly provided by PSi. A summary of the clinicopathological characteristics of these patient samples is given in Additional file 1: Table S1. The use of archived samples was approved by the ethics committee of the University of Heidelberg (206/2005). Patient informed consent (and a specific approval for this study) was not necessary, because the ethics approval covers the use of samples for retrospective analysis. The selection process was based on sample validity (RNA amount) and *ERBB2*, *ESR1*, *PGR*, and *MKI67* marker expression obtained using the MammaTyper®. Different histological subtypes such as ductal, lobular, tubular, and micropapillary breast carcinomas were included in the cohort. To encompass the entire range of clinically anticipated expression levels, six samples were selected for each marker equally distributed from the lowest to the highest expression value (sextiles), including values close to the cutoff. From among the selected 24 FFPE tissue samples, 8 were chosen for preparation of the RNA pools; the other 16 were used for preparation of the FFPE tissue sections. Nineteen consecutive 10-μm sections were prepared per paraffin block, and homogeneity of the sections was ensured in a prequalification process by MammaTyper® analysis of the first, middle, and last sections (Additional file 2: Figure S1).

### RNA extraction

Total RNA was purified from 10-μm FFPE tissue sections using the paramagnetic particle-based RNXtract® RNA Extraction Kit (reference 90040; BioNTech Diagnostics GmbH) according to the manufacturer’s instructions. The RNXtract® kit has been validated as a preanalytical RNA extraction method for the MammaTyper® by the manufacturer.

### MammaTyper® test

The MammaTyper® (reference 90020; BioNTech Diagnostics GmbH) is a molecular in vitro diagnostic RT-qPCR test for the quantitative detection of the mRNA expression status of the genes *ERBB2*, *ESR1*, *PGR*, and *MKI67* in human FFPE breast cancer tissue from resection or core needle biopsies with at least 20% tumor cell content using whole-tissue sections without macrodissection. Primary analysis outputs are the normalized, quantitative single-marker results given as 40^−∆∆Cq^ (quantification cycle) values on a continuous scale [21]. The test also provides the status of each marker as a binary category (positive or negative) based on clinically validated marker- and device-specific cutoff values. The combination of the four binary single-marker results can be further translated into the molecular subtype of the given breast cancer sample according to the St. Gallen classification [2] (i.e. luminal A-like, luminal B-like [HER2-negative], luminal B-like [HER2-positive], HER2-positive [nonluminal], and triple-negative [ductal]). The MammaTyper® test was performed according to the Instructions for Use 150528-90020 revision 3.0, applying the following cutoffs for the LightCycler® 480 II instrument [40^−∆∆Cq^]: *ERBB2* = 41.10, *ESR1* = 38.00, *PGR* = 35.50, and *MKI67* = 35.50.

### Statistical analysis

The results were analyzed according to a predefined statistical analysis plan using SAS version 9.4 software (SAS Institute, Cary, NC, USA). The number of measurements (sample size) of the study was determined using simulations to achieve predefined levels of uncertainty using results of a previous method validation [21]. On each study day, exported raw C_q_ values were directly transferred by the operator to the statistician. To reflect a realistic estimate of the test precision, statistical outliers were not excluded from the analyses.

The precision of the quantitative single-marker assessments (40^−∆∆Cq^ values) was estimated by a random effects model II analysis of variance (ANOVA) with site as a random factor [20]. Because the variability does not depend on 40^−∆∆Cq^ values, the sample was also included as a (trivial) random factor in the model, which allows averaging of the variance components over the samples:

1. The intermediate precision referring to *interrun/day SD* is obtained as the residual SD in the ANOVA.
2. The reproducibility was calculated as the intersite SD summarizing the condition of different sites, operators, and instruments. The total SD is also presented, calculated as the square root of the sum of residual and intersite variance components. Because the total SD is the precision as experienced in clinical practice, we decided to report this parameter as the main result as a conservative approach.
3. The variance introduced by a different MammaTyper® lot obtained in a separate experiment was given as the interlot SD.
4. Agreement of the categorical marker results and the breast cancer biological subtypes across all sites was evaluated using Fleiss’ kappa statistics [22]. According to the method of Landis and Koch [23], the strength of the agreement was defined as follows: kappa < 0.00 = poor, 0.00–0.20 = slight, 0.21–0.40 = fair, 0.41–0.60 = moderate, 0.61–0.80 substantial, and 0.81–1.00 = almost perfect.
5. The ICC was estimated for the continuous scaled quantitative marker results and was used to evaluate the reproducibility and intermediate precision in relationship with the intersample variance using the approach proposed by Eliasziw et al. [24]. Thus, and different from the kappa statistic, the ICC determines the agreement of measured quantitative values over the whole measurement range, independent of any cutoff point [25]. The agreement is generally interpreted as follows: ICC < 0.40 = poor, 0.40–0.74 = fair to good, and 0.75–1.00 = excellent [26]. More stringent thresholds were recommended by Kirkegaard et al. [25] for IHC assessments, with an ICC level of 0.7 regarded as the minimum acceptable standard, 0.8 as good, and ≥0.9 as excellent. The latter thresholds were applied in this study.

In a final analysis, kappa and ICC values were simulated in a larger sample cohort using quantitative data from 769 breast cancer cases of the FinHer trial that had been measured previously by MammaTyper® [27]. ICC values were calculated using the intersample variance of the larger cohort along with the intersite and residual variance of the present study. To estimate the kappa values for this cohort, 1000 simulated pairs of datasets were created by adding random noise to the 40^−∆∆Cq^ values according to the marker-specific total variance observed in this study. For each pair (2 × 769 values), kappa values for binary marker results and subtypes were calculated, resulting in 1000 kappa values, of which the median kappa as well as the 2.5% and 97.5% percentiles are reported.

## Results

### Intermediate precision

**Fig. 2:**
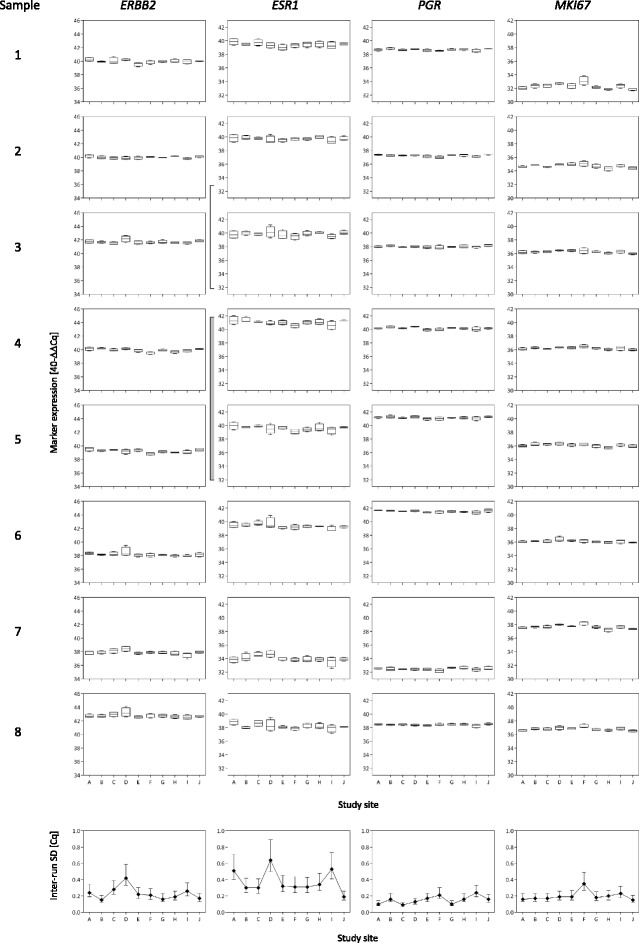
Box plots depicting inter- and intrasite reproducibility. The box plots represent the distribution of the four MammaTyper® measurements of the eight centrally extracted RNA samples for each marker at the ten different study sites A–J (study arm 1). The box plots indicate the median 40^−∆∆Cq^ values by the *horizontal line* dividing the boxes, the first and third quartiles by the *lower* and *upper borders* of the boxes, and the minimum and maximum values by the *whiskers*. The graphs at the *bottom* show the interrun SD over all samples for each marker at the respective site, as well as the corresponding 95% CI. *ERBB2* (HER2) Human epidermal growth factor receptor 2, *ESR1* (ER) Estrogen receptor, *MKI67* Marker of proliferation Ki67, *PGR* (PgR) Progesterone receptor

On the basis of MammaTyper® measurements of study arm 1 (Fig. 1), quantitative single-marker results were obtained as 40^−∆∆Cq^ values for *ERBB2*, *ESR1*, *PGR*, and *MKI67* and are presented in Fig. 2 as box plots for each marker, sample, and study site. The intermediate precision for each marker at the individual site was computed over all samples, presented as interrun SD (Fig. 2, graphs at the *bottom*).

### Intersite reproducibility

As indicated by the side-by-side box plots in Fig. 2, the 40^−∆∆Cq^ quantitative single-marker results of each individual sample were highly consistent across all ten study sites. The total SD of the measurements of the eight centrally extracted RNA samples (samples 1–8) was as low as 0.18 C_q_ for *PGR*, 0.29 C_q_ for *ERBB2* and *MKI67*, and 0.44 C_q_ for *ESR1* (Table 1, *upper panel*). As demonstrated by the variance component analysis, the factor site (intersite SD) had less impact on the total imprecision (total SD) than the interrun/day variability within one laboratory (residual SD) (Table 1, *upper panel*).

**Table 1:** Intersite reproducibility of centrally (samples 1–8) and locally (samples 9–24) extracted RNA samples as total SD, as well as intersite and residual SD (quantification cycle values)

	Analyte	Intersite		Residual		Total
		SD	95% CI	SD	95% CI	SD
RNA pools Samples 1–8	*ERBB2*	0.16	0.12–0.21	0.24	0.22–0.27	0.29
	*ESR1*	0.18	0.13–0.29	0.40	0.37–0.44	0.44
	*PGR*	0.09	0.07–0.13	0.16	0.15–0.17	0.18
	*MKI67*	0.21	0.17–0.26	0.21	0.19–0.23	0.29
Self-extracted RNAs Samples 9–24	*ERBB2*	0.22	0.19–0.26	0.23	0.21–0.24	0.31
	*ESR1*	0.25	0.20–0.31	0.36	0.34–0.39	0.44
	*PGR*	0.12	0.10–0.16	0.19	0.18–0.20	0.23
	*MKI67*	0.16	0.14–0.20	0.19	0.18–0.21	0.25
RNA pools and self-extracted RNAs Samples 1–24	*ERBB2*	0.20	0.17–0.23	0.23	0.22–0.25	0.31
	*ESR1*	0.22	0.19–0.27	0.38	0.36–0.40	0.44
	*PGR*	0.11	0.10–0.14	0.18	0.17–0.19	0.21
	*MKI67*	0.18	0.16–0.21	0.20	0.19–0.21	0.27

*Abbreviations: ERBB2* (HER2) Human epidermal growth factor receptor 2, *ESR1* (ER) Estrogen receptor, *MKI67* Marker of proliferation Ki67, *PGR* (PgR) Progesterone receptor

### Intersite reproducibility including preanalytical variances

**Fig. 3:**
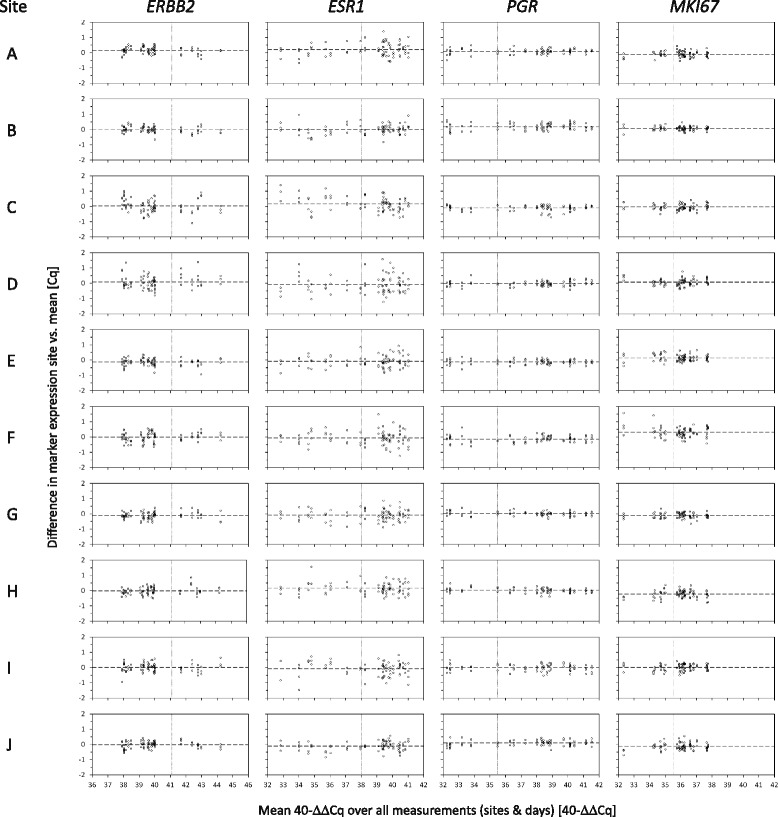
Bland-Altman plots representing inter- and intrasite reproducibility. Bland-Altman plots show the differences of the measured 40^−∆∆Cq^ values per sample, site, and marker (*y*-axis) against the respective mean over all measurements (sites and days) (*x*-axis). The 24 samples are represented by either 4 or 3 measurements for the centrally extracted (study arm 1) or self-extracted (study arm 2) RNA samples, respectively. The average deviation of measurements at the respective site is indicated by the *dashed horizontal line*. The *continuous vertical line* indicates the marker-specific cutoff. *ERBB2* (HER2) Human epidermal growth factor receptor 2, *ESR1* (ER) Estrogen receptor, *MKI67* Marker of proliferation Ki67, *PGR* (PgR) Progesterone receptor. **a**-**j** correspond to the ten study sites

The total variance of marker results (40^−∆∆Cq^ values) in the self-extracted samples (study arm 2, samples 9–24) was almost identical to the variance seen for the RNA pool aliquots (samples 1–8) (Table 1, *middle panel*). There was no additional variance or bias introduced by RNA extraction at local sites. Therefore, the intersite reproducibility was again computed on the whole sample set (samples 1–24), leading to a similar approximation of the total variability of single-marker 40^−∆∆Cq^ assessments with SDs between 0.21 and 0.44 C_q_ (Table 1, *lower panel*). Performing the analysis of the eight RNA pool samples with a different MammaTyper® lot resulted in comparable quantitative values (Additional file 3: Figure S2). The interlot SD was almost completely covered by the existing interrun/day variability (residual SD), and its impact on the total variance was negligible (Additional file 4: Table S2). Individual laboratory deviations for all samples are also shown with Bland-Altman plots (Fig. 3). The average deviation at the respective site was in all cases close to zero, with values ranging from C_q_ −0.13 to 0.16 for *ERBB2*, −0.11 to 0.20 for *ESR1*, −0.15 to 0.19 for *PGR*, and −0.22 to 0.31 for *MKI67*.

### Binary single-marker and subtype agreement

The binary single-marker results (positive/negative) for all measurements at the ten sites are displayed as counts for each sample in Table 2, revealing a very high concordance. The 24 samples showed 100% concordance for *ERBB2* and for *PGR* and *ESR1* an equivocal assignment in only one and two samples, respectively. These cases exhibited a marker expression level near the cutoff, as indicated by the distance to cutoff value (Table 2). This also explained the divergent measurements seen for *MKI67*, which biologically exhibits more samples near the cutoff because of its continuous distribution [28]. Nevertheless, *MKI67* showed a high agreement because for most discrepant cases only 1 of 30 determinations was classified differently (Table 2). Calculating the overall agreement of the categorical marker assessments resulted in kappa values of 1.00, 0.91, 0.94, and 0.94 for *ERBB2*, *ESR1*, *PGR*, and *MKI67*, respectively. Corresponding subtype assessments resulted in an almost perfect agreement with a kappa value of 0.90 (Table 3). Discrepancies were observed for example for the luminal A-like and luminal B-like (HER2-negative) subtype, where discrimination using St. Gallen guidelines relies on *MKI67* marker expression [2], which for the discrepant cases was very close to the cutoff (as described above).

**Table 2:** Binary single-marker agreements as positive/negative counts, and corresponding kappa values (distance of mean 40^−∆∆Cq^ value to marker-specific cutoff is shown for each sample)

	*ERBB2*			*ESR1*			*PGR*			*MKI67*		
Sample	Counts		Distance to cutoff (C_q_)	Counts		Distance to cutoff (C_q_)	Counts		Distance to cutoff (C_q_)	Counts		Distance to cutoff (C_q_)
	Negative	Positive		Negative	Positive		Negative	Positive		Negative	Positive	
1	**40**	0	−1.1	0	**40**	1.5	0	**40**	3.2	**40**	0	−3.2
2	**40**	0	−1.1	0	**40**	1.7	0	**40**	1.8	**40**	0	−0.8
3	0	**40**	0.6	0	**40**	1.9	0	**40**	2.5	0	**40**	0.8
4	**40**	0	−1.2	0	**40**	3.0	0	**40**	4.6	0	**40**	0.7
5	**40**	0	−1.9	0	**40**	1.6	0	**40**	5.6	0	**40**	0.6
6	**40**	0	−2.9	0	**40**	1.4	0	**40**	6.0	0	**40**	0.6
7	**40**	0	−3.2	**40**	0	−4.0	**40**	0	−3.0	0	**40**	2.2
8	0	**40**	1.7	**12**	**28**	0.2	0	**40**	2.9	0	**40**	1.3
9	0	**30**	1.3	**30**	0	−3.2	**30**	0	−2.3	0	**30**	1.1
10	0	**30**	3.1	**30**	0	−2.0	**30**	0	−1.7	0	**30**	1.5
11	**30**	0	−1.6	0	**30**	2.1	0	**30**	4.9	**4**	**26**	0.2
12	**30**	0	−1.8	0	**30**	2.7	0	**30**	4.7	0	**30**	1.1
13	**30**	0	−1.8	0	**30**	2.4	0	**30**	4.6	**1**	**29**	0.4
14	**30**	0	−2.0	0	**30**	1.4	0	**30**	3.2	**30**	0	−0.8
15	**30**	0	−1.3	0	**30**	1.1	0	**30**	1.0	**29**	**1**	−1.2
16	**29**	0	−1.5	0	**29**	2.5	0	**29**	2.9	0	**29**	0.7
17	**30**	0	−1.1	0	**30**	1.4	0	**30**	0.8	0	**30**	1.1
18	**30**	0	−1.3	0	**30**	2.5	0	**30**	4.2	**30**	0	−1.2
19	**30**	0	−1.5	0	**30**	2.8	0	**30**	2.8	**1**	**29**	0.4
20	**30**	0	−2.6	**30**	0	−0.9	0	**30**	3.3	**29**	**1**	−0.6
21	**30**	0	−2.8	**20**	**10**	0.0	0	**30**	3.4	**29**	**1**	−0.5
22	**30**	0	−3.1	**30**	0	−3.4	**30**	0	−3.1	0	**30**	2.1
23	**30**	0	−3.1	**30**	0	−2.3	**16**	**14**	0.0	0	**30**	2.2
24	0	**30**	1.9	**30**	0	−5.2	**30**	0	−3.3	**1**	**29**	0.4
kappa Statistic	1.00			0.91			0.94			0.94		

*Abbreviations: ERBB2* (HER2) Human epidermal growth factor receptor 2, *ESR1* (ER) Estrogen receptor, *MKI67* Marker of proliferation Ki67, *PGR* (PgR) Progesterone receptor

Boldface values represent summary of measurements

**Table 3:** Subtype agreement as counts, and corresponding kappa value

Sample	Not defined	HER2-positive (nonluminal)	Luminal B-like (HER2-positive)	Luminal B-like (HER2-negative)	Triple-negative (ductal)	Luminal A-like
1	0	0	0	0	0	**40**
2	0	0	0	0	0	**40**
3	0	0	**40**	0	0	0
4	0	0	0	**40**	0	0
5	0	0	0	**40**	0	0
6	0	0	0	**40**	0	0
7	0	0	0	0	**40**	0
8	**12**	0	**28**	0	0	0
9	0	**30**	0	0	0	0
10	0	**30**	0	0	0	0
11	0	0	0	**26**	0	**4**
12	0	0	0	**30**	0	0
13	0	0	0	**29**	0	**1**
14	0	0	0	0	0	**30**
15	0	0	0	**1**	0	**29**
16	0	0	0	**29**	0	0
17	0	0	0	**30**	0	0
18	0	0	0	0	0	**30**
19	0	0	0	**29**	0	**1**
20	**30**	0	0	0	0	0
21	**20**	0	0	0	0	**10**
22	0	0	0	0	**30**	0
23	**14**	0	0	0	**16**	0
24	0	**30**	0	0	0	0
kappa Statistic	0.90					

*Abbreviations: HER2* Human epidermal growth factor receptor 2

Boldface values represent summary of measurements

### Intra- and interclass correlation

ICC estimates of all markers were between 0.976 and 0.996 for the intralaboratory assessment (ICC_intra), and between 0.980 and 0.998 for the intersite reproducibility (ICC_inter) (Table 4, *upper panel*), reflecting excellent agreement of the quantitative data. To exclude any effect of the sample selection on ICC results, the ICCs were again computed using the intersample variance observed in the 769 breast cancer cases of the FinHer trial [27] along with the intersite and residual variance extracted from the present reproducibility study. The marker-specific ICCs in this analysis were nearly identical (Table 4, *lower panel*).

**Table 4:** Interclass correlation coefficient based on depicted variance components, and summary of kappa values

Analyte	Intersite variance	Intersample variance	Residual variance	ICC_intra	ICC_inter	kappa Statistic	
*ERBB2*	0.039	2.886	0.054	**0.982**	**0.987**	**1.00**	
*ESR1*	0.049	5.889	0.144	**0.976**	**0.992**	**0.91**	
*PGR*	0.013	8.279	0.031	**0.996**	**0.998**	**0.94**	
*MKI67*	0.032	1.565	0.039	**0.976**	**0.980**	**0.94**	
Subtype	N/A					**0.90**	
Analyte	Intersite variance (present study)	Intersample variance (FinHer data)	Residual variance (present study)	ICC_intra	ICC_inter	kappa Statistic (median)	2.5–97.5% percentile
*ERBB2*	0.039	3.723	0.054	**0.986**	**0.990**	**0.95**	0.92–0.97
*ESR1*	0.049	5.941	0.144	**0.977**	**0.992**	**0.97**	0.96–0.99
*PGR*	0.013	7.461	0.031	**0.996**	**0.998**	**0.97**	0.96–0.99
*MKI67*	0.032	1.739	0.039	**0.978**	**0.982**	**0.85**	0.82–0.88
Subtype	N/A					**0.91**	0.89-0.93

*Abbreviations: ERBB2* (HER2) Human epidermal growth factor receptor 2, *ESR1* (ER) Estrogen receptor, *MKI67* Marker of proliferation Ki67, *PGR* (PgR) Progesterone receptor, *N/A* Not applicable

*Note*: Intersite variance and residual variance are squares of SD presented in lower part of Table 1. The *lower panel* of this table is a simulated analysis using the intersample variance of data from the FinHer trial cohort (described in detail in the text)

Boldface values highlight ICC and kappa statistic

Finally, the kappa values were simulated in this larger sample cohort and were found to be comparable to the ones observed in the smaller sample size of the present study (Table 4, *lower panel*).

## Discussion

This study addressed the question whether the recently developed molecular in vitro diagnostic MammaTyper® test could improve the reproducibility of the assessment of the four key routine breast cancer biomarkers *ERBB2* (HER2), *ESR1* (ER), *PGR* (PgR), and *MKI67* (Ki67). The routine diagnostic assessment of these markers, as well as the corresponding subtyping, is currently performed by semiquantitative IHC and FISH, CISH, and SISH assays [1–4]. IHC assays suffer from considerable inter- and intralaboratory variability, which particularly applies to the assessment of the valuable biomarker Ki67 [8, 9, 15, 16]. Therefore, it is of importance that new technologies carrying the potential for more accurate, reliable, and precise analysis of Ki67 expression are brought under consideration to overcome the persisting inconsistencies [17, 18].

In this multicenter study, we demonstrated that MammaTyper® shows excellent inter- and intralaboratory precision, both for the continuous quantitative single-marker measurements (40^−∆∆Cq^ values) and for the categorical positive/negative status and the breast cancer molecular subtype classification. These data therefore confirm the high analytical performance of the MammaTyper® that was previously reported in the original technical validation of the test [21] but was shown in this study in a more comprehensive and challenging methodological setting. In our study, ten different laboratories were able to generate consistent and highly concordant test results after an initial training and a relatively short familiarization period. Overall, the test results were found to be independent of the preanalytical process and not influenced by the MammaTyper® lot. The source of imprecision of the quantitative measurements was related mainly to the general run-to-run variability rather than to the variance introduced by different laboratories. These observations were in line with the original technical validation report [21].

The ICC values for all markers were above 0.976, which signifies excellent agreement of the quantitative data and suggests improved inter- and intrasite reproducibility achieved with qPCR compared with what has been documented previously for IHC [9, 16]. In studies on IHC reproducibility, the intersite agreement for Ki67 IHC displays an ICC of 0.59, which is below the minimum acceptable standard proposed by Kirkegaard et al. [9, 25]. Even when standardized Ki67 scoring methods on centrally stained histological slides were tested, the interobserver reliability on resection specimens reached ICCs of only 0.40 to 0.74, with kappa values ranging from 0.29 to 0.58 [16]. Only training and precise calibration resulted in better Ki67 assessment on centrally stained tissue microarray slides (ICC 0.94) or centrally stained core-cut biopsies using a standardized scoring method (ICC 0.87), but this process is difficult to implement in routine clinical practice, and clinically important discrepancies persisted in the critical range of 10% to 20% Ki67-positive nuclei staining [15, 29]. The challenges in the standardization of Ki67 assessment include the variability in the selection of the tumor areas to be assessed, the technique used for nuclei counting, and the dilemma of the numerical cutoff for positivity, especially for large tissue sections [8, 9, 15, 16]. The highly promising reproducibility of the MammaTyper® was confirmed by a simulated analysis using MammaTyper® data obtained from 769 samples from the FinHer trial cohort [27], verifying that the study samples were representative of the whole spectrum of routine clinical samples.

The high values of the various reproducibility metrics in the present study are a result of both the underlying high degree of standardization of the MammaTyper® test, which minimizes the main sources of variability, and the adaptation of a fully objective assessment method (i.e., qPCR). Thus, the MammaTyper® assay has the potential to overcome the substantial and varying rates of inter- and intraobserver variability that may occur with IHC. This applies especially in samples where high-quality IHC is not readily available for diagnostic purposes. Analytical validity, such as reproducibility, is a prerequisite for accurate diagnostics, and its formal evaluation is required along with a test’s clinical performance to allow conclusions on its potential use in clinical practice [17, 18]. In a previous clinical performance evaluation study, good concordance was shown between MammaTyper® single-marker assessments and IHC (or IHC/CISH for HER2), using 769 archived breast cancer cases available from the FinHer trial [27]. Only for *MKI67* mRNA expression was the correlation moderate, most likely because of the analytical restriction of Ki67 IHC. The multivariable analysis revealed that *MKI67* expression assessed by MammaTyper®, but not Ki67 IHC, was an independent predictor of distant disease-free survival (DDFS), indicating the superiority of MammaTyper® compared with IHC with respect to *MKI67*/Ki67 determination [27]. Furthermore, this clinical performance evaluation study demonstrated that the mRNA-based subtyping by MammaTyper® resulted in clinically meaningful prognostic and predictive information with regard to DDFS, overall survival, and response to taxane-based chemotherapy in the luminal B-like (HER2-negative) subtype [27]. These published data provide evidence for the clinical validity of the test, and additional clinical performance evaluation studies would help further strengthen its clinical value for routine applications. As is true of all molecular assays that use tissue homogenates, one may also consider low tumor cell content or high lymphocytic infiltration as potential sources of error. For MammaTyper®, a minimum tumor cell content of 20% was required to generate stable test results when compared with the paired macrodissected sample [21]. Adjacent nontumor tissue had no major influence on test results, likely because of the reduced metabolic activity and low RNA content in the surrounding tissue compared with the invasive tumor [30, 31]. As reported previously, nontumor components may have a stronger impact on multigene tests that analyze a recurrence score based on genes with partially notable expression in normal tissue [32, 33]. Nevertheless, further validations of MammaTyper® on samples with problematic characteristics (i.e., varying amounts of ductal carcinoma in situ and lymphocytic infiltrates) should be envisaged.

The reliable quantification of single-marker expression by MammaTyper® has the potential to become part of a predictive marker panel in breast cancer diagnostics with further refined implications for clinical management. The expression of the single markers *ERBB2*, *ESR1*, *PGR*, and *MKI67* is obtained on a continuous scale covering a much broader dynamic range (up to 5 orders of magnitude) than can be achieved by IHC (up to 2 orders of magnitude; 0–100% positive cells or H-score 0–300) [21, 34]. An ongoing challenge in clinical management is the decision whether to treat patients with luminal breast cancer with systemic chemotherapy when other clinicopathological factors, such as nodal status, are not decisive [17]. Various multigene assays address exactly this diagnostic dilemma. Oncotype DX® (Genomic Health, Redwood City, CA, USA), MammaPrint® (Agendia, Irvine, CA, USA), EndoPredict® (Myriad Genetics, Salt Lake City, UT, USA), and Prosigna® (NanoString Technologies, Seattle, WA, USA) provide risk scores with prognostic information for distant recurrence in patients with luminal A-like and luminal B-like (HER2-negative) breast cancers. High and low risk scores, even though they are mainly prognostic, are frequently used to decide for or against chemotherapy in the ER/PgR-positive, HER2-negative subgroup [17]. Limitations exist, however, because the appropriate course of action remains unclear for patients with intermediate risk cancers or for node-positive patients. Prospective studies on multigene tests in breast cancer showing predictive values are limited [35–37]. The randomized phase III Microarray in Node-Negative and 1 to 3 Positive Lymph Node Disease May Avoid Chemotherapy (MINDACT) trial recently demonstrated that chemotherapy can be omitted in around 46% of clinically high-risk cases with low MammaPrint® scores [37]. Similarly, the recurrence score measured with Oncoytpe DX® in the prospective randomized phase III Plan B study demonstrated excellent 3-year survival by omitting chemotherapy in clinically high-risk but recurrence score low-risk cases [35]. Further prospective studies on Oncotype DX® testing revealed excellent 5-year survival (98%) in low-risk score cases treated with hormone therapy alone [36, 38]. However, these tests come with high costs. In addition, some of these tests require sending the samples to a central laboratory, such as Oncotype DX® and MammaPrint®. In this respect, it is interesting that a similar score, namely the immunohistochemical 4 (IHC4) score, with comparable prognostic value was generated by using just the four IHC markers HER2, ER, PgR, and Ki67 [39, 40]. However, insufficient standardization and considerable interlaboratory variability of IHC suggest that the IHC4 algorithm cannot easily be transferred to other laboratories, although it was successfully validated in an independent study cohort [41]. On the basis of data obtained in this study, MammaTyper® could provide a highly reproducible and reliable assessment of these four markers. It is tempting to suggest that in the future a similar approach might be applicable for the MammaTyper® to generate additional prognostic information to guide personalized treatment options at much lower cost than multigene expression tests.

## Conclusions

The MammaTyper® test has the potential to improve the quality of primary breast cancer molecular diagnostics. The test showed reliable reproducibility in the quantitative assessment of the single markers *ERBB2*, *ESR1*, *PGR*, and *MKI67*, as well as in the subtype determination, and thereby overcomes the variability known on the basis of diagnostic experience with IHC. The low intersite and intrasite variance of the MammaTyper® test enables pathology institutions to perform this assay in-house and integrate the technology into routine diagnostic services. However, additional clinical performance evaluation studies in larger cohorts are necessary to confirm the clinical utility of the test and to extract further predictive information for more personalized clinical management of patients with breast cancer.

## Additional files

Additional file 1: Table S1. Clinicopathological characteristics of the breast cancer patient samples used in this study, including HER2, ER, PgR, and Ki67 marker status. (DOC 61 kb)

Additional file 2: Figure S1. Prequalification of FFPE sections for study arm 2. Presented are 40-∆∆C_q_ values of section numbers 1, 10, and 19 for each marker to confirm homogeneous expression between sections. (PDF 553 kb)

Additional file 3: Figure S2. Box plots depicting interlot reproducibility. The box plots represent the distribution of the four MammaTyper® measurements of the eight RNA pool samples using two different MammaTyper® lots at one site. The box plots indicate the median 40^−∆∆Cq^ values by the horizontal line dividing the boxes, the first and third quartiles by the lower and upper border of the boxes, and the minimum and maximum values by the whiskers. (PDF 1359 kb)

Additional file 4: Table S2. Interlot reproducibility of centrally extracted RNA samples (samples 1–8) as total SD, and the variance components interlot and residual SD (in C_q_). (DOC 31 kb)

## Abbreviations

ANOVA: Analysis of variance
CISH: Chromogenic in situ hybridization
C_q_: Quantification cycle
DDFS: Distant disease-free survival
*ERBB2* (HER2): Human epidermal growth factor receptor 2
*ESR1* (ER): Estrogen receptor
FFPE: Formalin-fixed, paraffin-embedded
FISH: Fluorescence in situ hybridization
ICC: Interclass correlation coefficient
IHC: Immunohistochemistry
IHC4: Immunohistochemical 4 score
*MKI67*: Marker of proliferation Ki67
mRNA: Messenger RNA
*PGR* (PgR): Progesterone receptor
RT-qPCR: Reverse transcription-quantitative real-time polymerase chain reaction
SISH: Silver in situ hybridization
