# Supplementary material for: An international reproducibility study validating quantitative determination of ERBB2, ESR1, PGR, and MKI67 mRNA in breast cancer using MammaTyper®
Source: Breast Cancer Res. 2017 May 11;19:55. doi: 10.1186/s13058-017-0848-z (PMC5426065; doi:10.1186/s13058-017-0848-z)
Supplement: Supplementary file 1 — Clinicopathological characteristics of the breast cancer patient samples used in this study, including HER2, ER, PgR, and Ki67 marker status. (DOC 61 kb) [file 13058_2017_848_MOESM1_ESM.doc]

**Additional file 1: Table S1** Clinico-pathological characteristics of the breast cancer patient samples used in this study, including the HER2, ER, PgR and Ki67 marker status

| **Sample** | **Age [years]** | **Surgery** | **Tumor size [cm]** | **Histological type** | **Grade** | **pT** | **pN** | **HER2** | **ER [%]** | **PgR [%]** | **Ki67 [%]** |
| --- | --- | --- | --- | --- | --- | --- | --- | --- | --- | --- | --- |
| **1** | 50 | BCT | 7.5 | ILC | 1 | 1b | 0 | 0 | 40 | 100 | <5 |
| **2** | 49 | BCT | 7 | CRIB | 1 | 1c | 0 | 0 | 90 | 90 | <5 |
| **3** | 49 | ME | 17 | TUB | 1 | 1c | 0 | 0 | 90 | 90 | 10 |
| **4** | 44 | ME | 13 | IDC | 2 | 2 | 0 | 0 | 100 | 100 | 5 |
| **5** | 43 | BCT | 3.5 | IDC | 1 | 1c | 0 | 0 | 100 | 100 | 10 |
| **6** | 50 | BCT | 6 | IDC | 2 | 1c | 0 | 0 | 100 | 100 | 5 |
| **7** | 75 | BCT | 6 | IDC | 3 | 2 | 0 | 0 | 0 | 0 | 80 |
| **8** | 89 | ME | 22 | IDC | 2 | 1c | 0 | 3+ | 100 | 90 | 60 |
| **9** | 58 | BCT | 7 | IDC | 3 | 2 | 3a | 3+ | 0 | 0 | 40 |
| **10** | 64 | BCT | 9 | IDC | 3 | 1b | 0 | 3+ | 0 | 0 | 40 |
| **11** | 49 | ME | 16 | TUB | 1 | 1b | 0 | 0 | 90 | 100 | 2 |
| **12** | 44 | ME | 18 | IDC | 2 | 1b | 0 | 0 | 90 | 90 | 20 |
| **13** | 47 | BCT | 6.7 | IDC | 2 | 1c | 0 | 1+ | 95 | 95 | 10 |
| **14** | 49 | ME | 14 | IDC | 2 | 1c | 1mi | 2+* | 90 | 90 | 5 |
| **15** | 59 | ME | 13.5 | ILC | 2 | 1c | 0 | 0 | 60 | 90 | 2 |
| **16** | 46 | BCT | 4.8 | IDC | 1 | 1c | 0 | 0 | 30 | 30 | 10 |
| **17** | 41 | ME | 20 | IMPC | 2 | 1b(m) | 0 | 1+ | 100 | 5 | 20 |
| **18** | 43 | ME | 20 | IDC | 2 | 1c | 0 | 1+ | 100 | 100 | 15 |
| **19** | 41 | BCT | 12.5 | IDC | 2 | 2 | 0 | 0 | 100 | 40 | 5 |
| **20** | 43 | BCT | 7 | IDC | 1 | 1c | 0 | 0 | 95 | 100 | 50 |
| **21** | 49 | BCT | 3 | IDC | 3 | 1c | 1a | 0 | 0 | 0 | 80 |
| **22** | 40 | ME | 28 | IDC | 3 | 3 | 3a | 0 | 0 | 0 | 40 |
| **23** | 34 | BCT | 6 | IDC | 3 | 1c | 0 | 0 | 0 | 0 | 90 |
| **24** | 79 | BCT | 4.5 | IDC | 2 | 2 | 0 | 3+ | 5 | 5 | 25 |

Abbreviations: BCT, breast conserving therapy; ME, mastectomy; IDC, Invasive Ductal Carcinoma; ILC, Invasive Lobular Carcinoma; TUB, Tubular carcinoma; CRIB, Cribriform carcinoma; IMPC, Invasive Micropapillary Carcinoma; pT, pathologic tumor stage; pN, pathologic nodal stage.
