# Supplementary figures and images for: An international reproducibility study validating quantitative determination of ERBB2, ESR1, PGR, and MKI67 mRNA in breast cancer using MammaTyper®
Source: Breast Cancer Res. 2017 May 11;19:55. doi: 10.1186/s13058-017-0848-z (PMC5426065; doi:10.1186/s13058-017-0848-z)

Figure S1

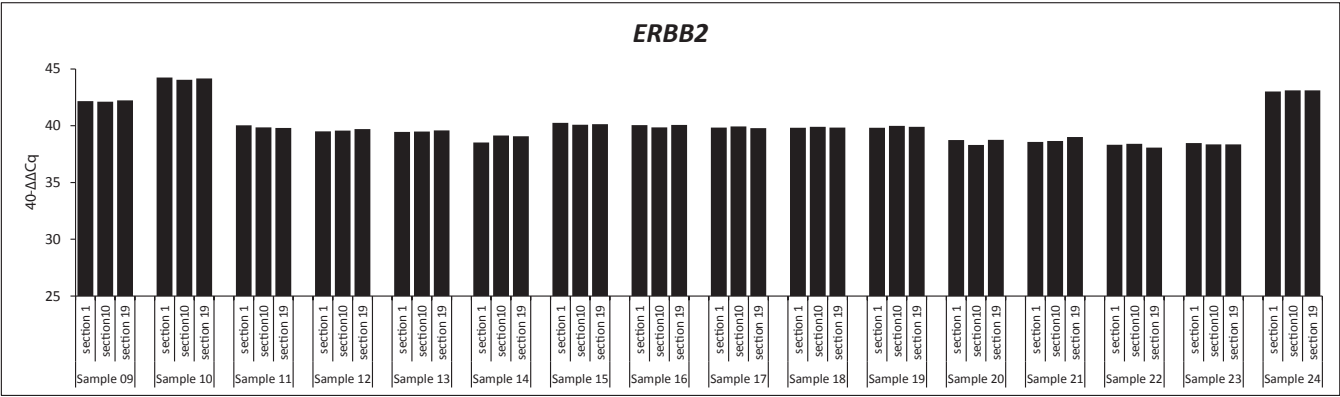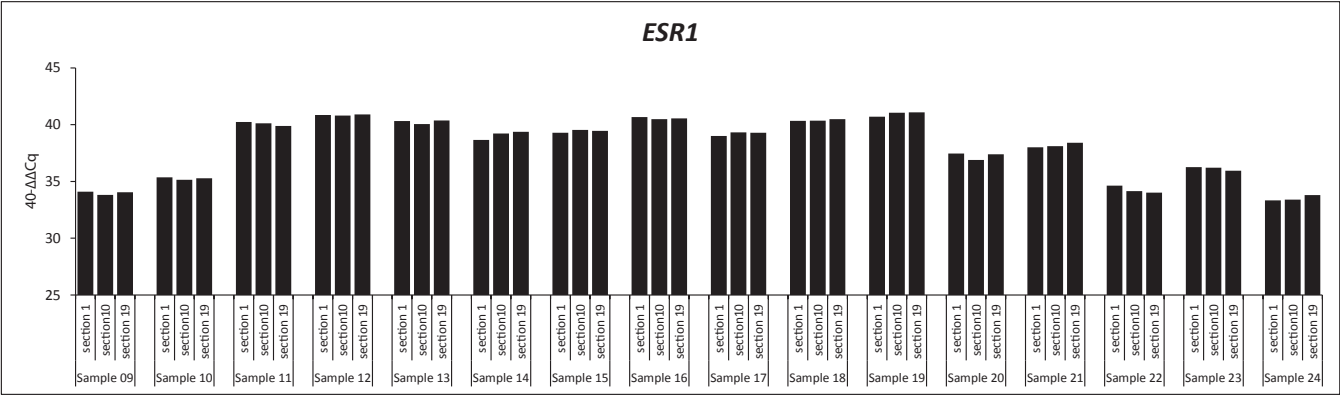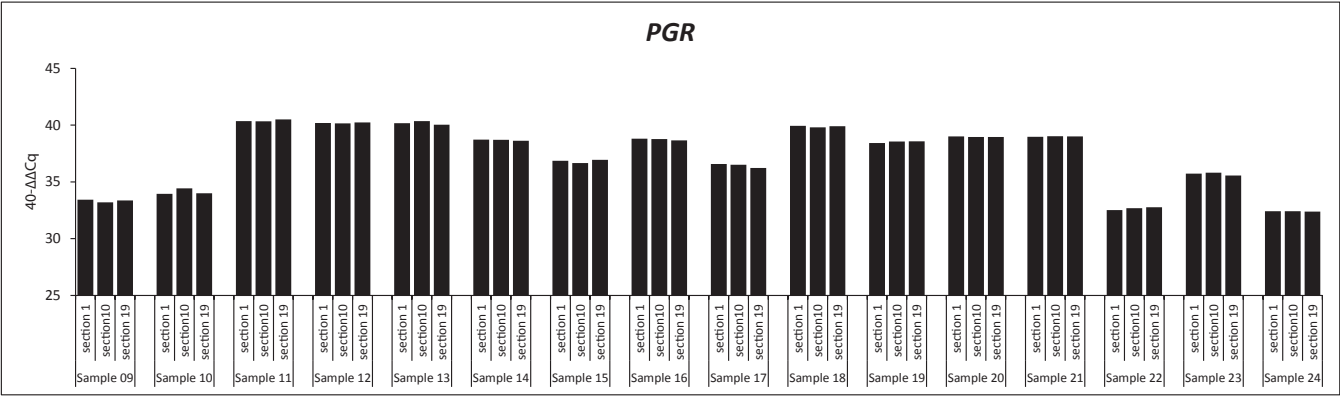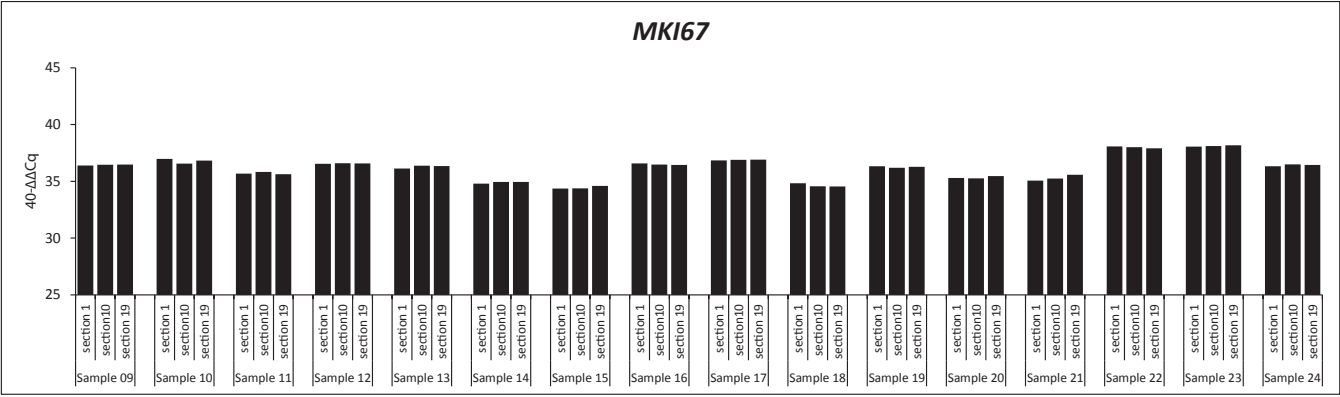

Supplement: Supplementary file 2 — Prequalification of FFPE sections for study arm 2. Presented are 40-∆∆Cq values of section numbers 1, 10, and 19 for each marker to confirm homogeneous expression between sections. (PDF 553 kb) [file 13058_2017_848_MOESM2_ESM.pdf]

Figure S2.

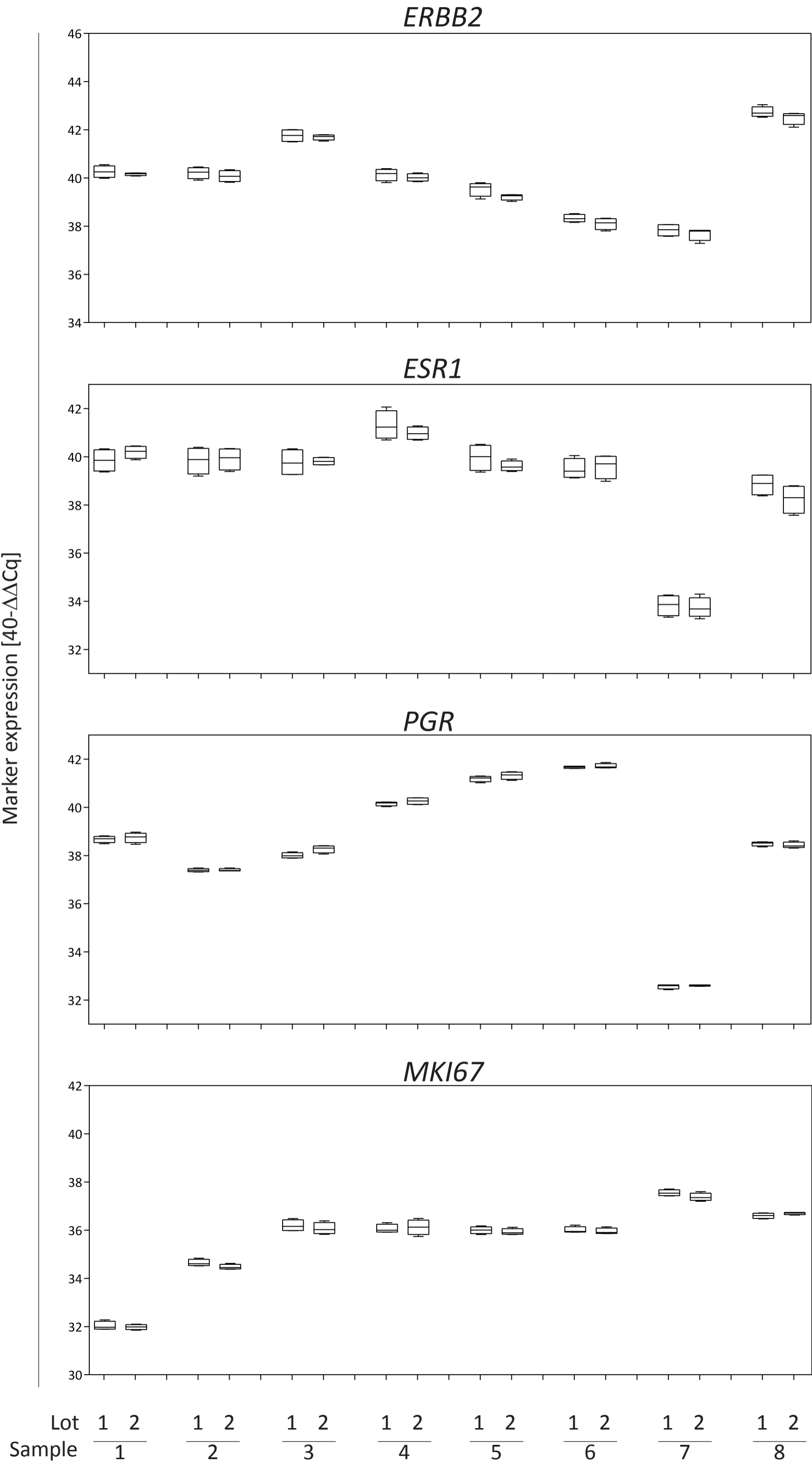

Supplement: Supplementary file 3 — Box plots depicting interlot reproducibility. The box plots represent the distribution of the four MammaTyper® measurements of the eight RNA pool samples using two different MammaTyper® lots at one site. The box plots indicate the median 40−∆∆Cq values by the horizontal line dividing the boxes, the first and third quartiles by the lower and upper border of the boxes, and the minimum and maximum values by the whiskers. (PDF 1359 kb) [file 13058_2017_848_MOESM3_ESM.pdf]
