# Supplementary material for: An international reproducibility study validating quantitative determination of ERBB2, ESR1, PGR, and MKI67 mRNA in breast cancer using MammaTyper®
Source: Breast Cancer Res. 2017 May 11;19:55. doi: 10.1186/s13058-017-0848-z (PMC5426065; doi:10.1186/s13058-017-0848-z)
Supplement: Supplementary file 4 — Interlot reproducibility of centrally extracted RNA samples (samples 1–8) as total SD, and the variance components interlot and residual SD (in Cq). (DOC 31 kb) [file 13058_2017_848_MOESM4_ESM.doc]

**Additional file 4: Table S2** Inter-lot reproducibility of centrally extracted RNA samples (Samples 1-8) as total SD, and the variance components inter-lot and residual SD (in Cq)

|  | **Analyte** | **Inter-lot** | | **Residual** | | **Total** |
| --- | --- | --- | --- | --- | --- | --- |
|  |  | **SD** | **95% CI** | **SD** | **95% CI** | **SD** |
| **RNA pools**  **Sample 1-8** | ***ERBB2*** | 0.08 | 0.03-4.63 | 0.22 | 0.18-0.27 | **0.23** |
|  | ***ESR1*** | 0.00* | NA | 0.45 | 0.38-0.55 | **0.45** |
|  | ***PGR*** | 0.06 | 0.03-0.37 | 0.12 | 0.10-0.15 | **0.13** |
|  | ***MKI67*** | 0.00* | NA | 0.17 | 0.14-0.20 | **0.17** |

* the inter-lot variance is completely explained (covered) by the residual variance
